# (4-Chloro­benzohydrazidato-κ^2^ *N*′,*O*)[2-(4-chloro­benzoyl­hydrazinyl­idene-κ^2^ *N* ^1^,*O*)-3-phenyl­propionato(2−)-κ*O* ^1^]oxidovanadium(V) methanol monosolvate

**DOI:** 10.1107/S1600536810045678

**Published:** 2010-11-13

**Authors:** Hon Wee Wong, Kong Mun Lo, Seik Weng Ng

**Affiliations:** a Department of Chemistry, University of Malaya, 50603 Kuala Lumpur, Malaysia

## Abstract

The V^V^ atom in the title compound, [V(C_7_H_6_ClN_2_O)(C_16_H_11_ClN_2_O_3_)O]·CH_3_OH, is *N*,*O*-chelated by the benzoyl­hydrazidate anion and *O*,*N*,*O*′-chelated by the (benzoyl­hydrazinyl­idene)propionate dianion. The distorted octa­hedral *trans*-N_2_O_4_ coordination geometry is completed by the vanadyl O atom. The mononuclear and solvent mol­ecules are linked by N—H⋯O and O—H⋯O hydrogen bonds about a center of inversion, generating a dimer.

## Related literature

For (benzohydrazidato)[2-(benzoyl­hydrazinyl­idene)pro­pio­nato)(2–)]oxidovanadium(V), see: Wong *et al.* (2009*a* ▶,*b* ▶).
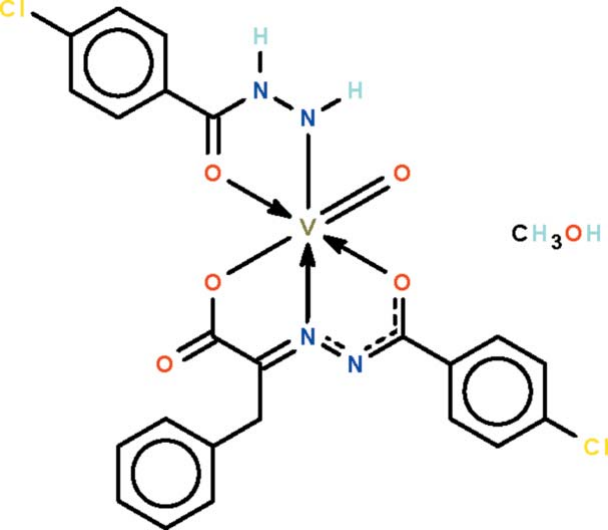


## Experimental

### 

#### Crystal data

[V(C_7_H_6_ClN_2_O)(C_16_H_11_ClN_2_O_3_)O]·CH_4_O
*M* *_r_* = 583.29
Triclinic, 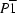

*a* = 8.3217 (4) Å
*b* = 11.2505 (6) Å
*c* = 15.5064 (8) Å
α = 109.4045 (7)°
β = 98.8890 (7)°
γ = 111.6936 (7)°
*V* = 1206.93 (11) Å^3^
*Z* = 2
Mo *K*α radiation
μ = 0.68 mm^−1^
*T* = 100 K
0.30 × 0.20 × 0.10 mm

#### Data collection

Bruker SMART APEX diffractometer
Absorption correction: multi-scan (*SADABS*; Sheldrick, 1996 ▶) *T*_min_ = 0.821, *T*_max_ = 0.935
15037 measured reflections
5517 independent reflections
4907 reflections with *I* > 2σ(*I*)
*R*_int_ = 0.021

#### Refinement

*R*[*F* ^2^ > 2σ(*F* ^2^)] = 0.038
*wR*(*F* ^2^) = 0.116
*S* = 1.03
5517 reflections
335 parameters
H-atom parameters constrained
Δρ_max_ = 0.54 e Å^−3^
Δρ_min_ = −0.85 e Å^−3^

### 

Data collection: *APEX2* (Bruker, 2009 ▶); cell refinement: *SAINT* (Bruker, 2009 ▶); data reduction: *SAINT*; program(s) used to solve structure: *SHELXS97* (Sheldrick, 2008 ▶); program(s) used to refine structure: *SHELXL97* (Sheldrick, 2008 ▶); molecular graphics: *X-SEED* (Barbour, 2001 ▶); software used to prepare material for publication: *publCIF* (Westrip, 2010 ▶).

## Supplementary Material

Crystal structure: contains datablocks global, I. DOI: 10.1107/S1600536810045678/hg2740sup1.cif

Structure factors: contains datablocks I. DOI: 10.1107/S1600536810045678/hg2740Isup2.hkl

Additional supplementary materials:  crystallographic information; 3D view; checkCIF report

## Figures and Tables

**Table 1:** Hydrogen-bond geometry (Å, °)

*D*—H⋯*A*	*D*—H	H⋯*A*	*D*⋯*A*	*D*—H⋯*A*
N3—H3⋯O1^i^	0.88	1.98	2.741 (2)	143
N4—H4⋯O6^i^	0.88	1.94	2.792 (3)	162
O6—H6⋯O2	0.84	2.27	2.908 (3)	133

Symmetry code: (i) 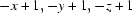.

**Fig. 1.**
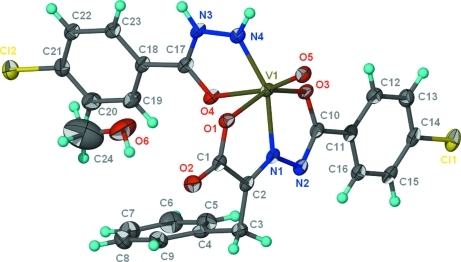
Thermal ellipsoid plot (Barbour, 2001) of VO(C7H6ClN2O)(C16H11ClN2O3).CH3OH at the 70% probability level. Hydrogen atoms are drawn as spheres of arbitrary radius.

## supplementary crystallographic information

### Comment

The reaction of vanadyl(IV) sulfate and the Schiff base that is synthesized by condensing a substituted benzhydrazine and a substituted pyruvic acid leads a vanadium(V) derivative of the Schiff base. However, another mole of the Schiff base is cleaved and the resulting benzhydrazine monoanion also chelates to the metal atom (Wong *et al.*, 2009*a*, 2009*b*). A similar product is isolated in the present study on the reaction of the Schiff base, 2-[4-chlorobenzoylhydrazono]-3-phenylpropionic acid so that the metal atom is chelated by two different ligands. The mononuclear mixed-ligand compound crystallizes as a monosolvate (Scheme I, Fig. 1). The vanadium(V) atom is *N*,*O*-chelated by the benzoylhydrazidate anion and *O*,*N*,*O*'-chelated by the (benzoylhydrazinylidene)propionate dianion; the terdentate chelate binds in a meridional mode. The octahedral *trans*-N_2_O_4_ coordination geometry is completed by the vanadyl O atom. The mononuclear and solvent molecules are linked by hydrogen bonds about a center-of-inversion to generate a hydrogen-bonded dimer.

### Experimental

2-[4-Chlorobenzoylhydrazono]-3-phenylpropionic acid prepared from the condensation reaction of 4-chlorobenzhydrazide and 3-phenylpyruvic acid. The compound (1.00 g, 3 mmol) and vanadyl sulfate (1.25 g, 1.5 mmol) in 50 ml of methanol for 5 h. Slow evaporation of the filtrate gave brownish orange crystals.

### Refinement

Carbon-, nitrogen- and oxygen-bound H-atoms were placed in calculated positions (C–H 0.95 to 0.99 Å, N–H 0.86 Å and O–H 0.84 Å) and were included in the refinement in the riding model approximation, with *U*(H) set to 1.2 to 1.5*U*(C).

### Crystal data

**Table:**

[V(C_7_H_6_ClN_2_O)(C_16_H_11_ClN_2_O_3_)O]·CH_4_O	*Z* = 2
*M**_r_* = 583.29	*F*(000) = 596
Triclinic, *P*1	*D*_x_ = 1.605 Mg m^−^^3^
Hall symbol: -P 1	Mo *K*α radiation, λ = 0.71073 Å
*a* = 8.3217 (4) Å	Cell parameters from 8039 reflections
*b* = 11.2505 (6) Å	θ = 2.6–28.3°
*c* = 15.5064 (8) Å	µ = 0.68 mm^−^^1^
α = 109.4045 (7)°	*T* = 100 K
β = 98.8890 (7)°	Block, brown
γ = 111.6936 (7)°	0.30 × 0.20 × 0.10 mm
*V* = 1206.93 (11) Å^3^	

### Data collection

**Table:**

Bruker SMART APEX diffractometer	5517 independent reflections
Radiation source: fine-focus sealed tube	4907 reflections with *I* > 2σ(*I*)
graphite	*R*_int_ = 0.021
ω scans	θ_max_ = 27.5°, θ_min_ = 2.2°
Absorption correction: multi-scan (*SADABS*; Sheldrick, 1996)	*h* = −10→10
*T*_min_ = 0.821, *T*_max_ = 0.935	*k* = −14→14
15037 measured reflections	*l* = −20→20

### Refinement

**Table:**

Refinement on *F*^2^	Primary atom site location: structure-invariant direct methods
Least-squares matrix: full	Secondary atom site location: difference Fourier map
*R*[*F*^2^ > 2σ(*F*^2^)] = 0.038	Hydrogen site location: inferred from neighbouring sites
*wR*(*F*^2^) = 0.116	H-atom parameters constrained
*S* = 1.03	*w* = 1/[σ^2^(*F*_o_^2^) + (0.0658*P*)^2^ + 1.2158*P*] where *P* = (*F*_o_^2^ + 2*F*_c_^2^)/3
5517 reflections	(Δ/σ)_max_ = 0.001
335 parameters	Δρ_max_ = 0.54 e Å^−^^3^
0 restraints	Δρ_min_ = −0.85 e Å^−^^3^

### Fractional atomic coordinates and isotropic or equivalent isotropic displacement parameters (Å^2^)

**Table:**

	*x*	*y*	*z*	*U*_iso_*/*U*_eq_	
V1	0.70290 (4)	0.70483 (4)	0.70065 (2)	0.01467 (10)	
Cl1	1.70732 (7)	1.31564 (6)	1.15657 (4)	0.02577 (14)	
Cl2	0.91329 (8)	0.00311 (6)	0.37080 (4)	0.02773 (14)	
O1	0.47185 (19)	0.53272 (15)	0.66919 (10)	0.0174 (3)	
O2	0.3293 (2)	0.35991 (16)	0.71185 (11)	0.0225 (3)	
O3	0.95256 (19)	0.84331 (15)	0.78583 (10)	0.0179 (3)	
O4	0.8120 (2)	0.54984 (16)	0.65625 (10)	0.0185 (3)	
O5	0.6190 (2)	0.81279 (16)	0.70892 (11)	0.0204 (3)	
O6	0.4172 (3)	0.2179 (3)	0.54539 (15)	0.0477 (5)	
H6	0.3470	0.2073	0.5787	0.072*	
N1	0.7353 (2)	0.66447 (18)	0.82203 (12)	0.0151 (3)	
N2	0.8951 (2)	0.75265 (18)	0.89745 (12)	0.0167 (3)	
N3	0.7294 (2)	0.56220 (19)	0.51836 (12)	0.0174 (3)	
H3	0.7125	0.5358	0.4562	0.021*	
N4	0.6962 (2)	0.66803 (18)	0.57236 (12)	0.0174 (3)	
H4	0.6710	0.7206	0.5467	0.021*	
C1	0.4566 (3)	0.4711 (2)	0.72789 (14)	0.0171 (4)	
C2	0.6167 (3)	0.5496 (2)	0.81905 (14)	0.0162 (4)	
C3	0.6358 (3)	0.4883 (2)	0.88969 (15)	0.0193 (4)	
H3A	0.5156	0.4400	0.8968	0.023*	
H3B	0.7195	0.5644	0.9535	0.023*	
C4	0.7112 (3)	0.3833 (2)	0.85310 (15)	0.0196 (4)	
C5	0.8986 (3)	0.4305 (3)	0.87107 (18)	0.0266 (5)	
H5	0.9795	0.5281	0.9077	0.032*	
C6	0.9674 (4)	0.3354 (3)	0.8356 (2)	0.0347 (6)	
H6A	1.0952	0.3683	0.8482	0.042*	
C7	0.8502 (4)	0.1930 (3)	0.7821 (2)	0.0364 (6)	
H7	0.8976	0.1281	0.7587	0.044*	
C8	0.6639 (4)	0.1455 (3)	0.76274 (19)	0.0333 (6)	
H8	0.5833	0.0479	0.7255	0.040*	
C9	0.5945 (3)	0.2402 (2)	0.79772 (17)	0.0262 (5)	
H9	0.4665	0.2071	0.7838	0.031*	
C10	0.9974 (3)	0.8461 (2)	0.87082 (14)	0.0162 (4)	
C11	1.1731 (3)	0.9611 (2)	0.94136 (14)	0.0172 (4)	
C12	1.2677 (3)	1.0766 (2)	0.92264 (15)	0.0202 (4)	
H12	1.2197	1.0797	0.8642	0.024*	
C13	1.4313 (3)	1.1865 (2)	0.98931 (16)	0.0216 (4)	
H13	1.4952	1.2659	0.9774	0.026*	
C14	1.4999 (3)	1.1790 (2)	1.07313 (15)	0.0196 (4)	
C15	1.4091 (3)	1.0649 (2)	1.09321 (15)	0.0200 (4)	
H15	1.4592	1.0613	1.1511	0.024*	
C16	1.2445 (3)	0.9567 (2)	1.02717 (15)	0.0184 (4)	
H16	1.1795	0.8789	1.0403	0.022*	
C17	0.7901 (3)	0.5014 (2)	0.56797 (14)	0.0172 (4)	
C18	0.8251 (3)	0.3815 (2)	0.51698 (15)	0.0173 (4)	
C19	0.8981 (3)	0.3266 (3)	0.57203 (16)	0.0249 (5)	
H19	0.9271	0.3685	0.6402	0.030*	
C20	0.9286 (3)	0.2114 (3)	0.52785 (17)	0.0272 (5)	
H20	0.9789	0.1740	0.5651	0.033*	
C21	0.8842 (3)	0.1515 (2)	0.42783 (16)	0.0209 (4)	
C22	0.8150 (3)	0.2059 (2)	0.37210 (15)	0.0187 (4)	
H22	0.7878	0.1646	0.3041	0.022*	
C23	0.7861 (3)	0.3220 (2)	0.41712 (14)	0.0173 (4)	
H23	0.7395	0.3610	0.3798	0.021*	
C24	0.4283 (7)	0.1015 (6)	0.5053 (4)	0.0873 (15)	
H24A	0.4884	0.1067	0.4561	0.131*	
H24B	0.3056	0.0221	0.4750	0.131*	
H24C	0.4994	0.0876	0.5543	0.131*	

### Atomic displacement parameters (Å^2^)

**Table:**

	*U*^11^	*U*^22^	*U*^33^	*U*^12^	*U*^13^	*U*^23^
V1	0.01538 (17)	0.01633 (18)	0.01099 (17)	0.00714 (14)	0.00334 (13)	0.00470 (13)
Cl1	0.0165 (2)	0.0231 (3)	0.0221 (3)	0.0044 (2)	0.0027 (2)	−0.0014 (2)
Cl2	0.0365 (3)	0.0239 (3)	0.0284 (3)	0.0188 (2)	0.0127 (2)	0.0106 (2)
O1	0.0156 (7)	0.0198 (7)	0.0140 (7)	0.0066 (6)	0.0026 (5)	0.0064 (6)
O2	0.0191 (7)	0.0212 (8)	0.0198 (7)	0.0039 (6)	0.0034 (6)	0.0075 (6)
O3	0.0175 (7)	0.0187 (7)	0.0119 (7)	0.0051 (6)	0.0024 (5)	0.0047 (6)
O4	0.0186 (7)	0.0222 (7)	0.0132 (7)	0.0093 (6)	0.0047 (5)	0.0057 (6)
O5	0.0229 (7)	0.0216 (7)	0.0164 (7)	0.0115 (6)	0.0054 (6)	0.0066 (6)
O6	0.0594 (14)	0.0624 (15)	0.0313 (11)	0.0334 (12)	0.0211 (10)	0.0214 (10)
N1	0.0156 (8)	0.0170 (8)	0.0116 (7)	0.0085 (7)	0.0040 (6)	0.0037 (6)
N2	0.0138 (8)	0.0178 (8)	0.0130 (8)	0.0056 (7)	0.0013 (6)	0.0034 (7)
N3	0.0173 (8)	0.0209 (9)	0.0112 (8)	0.0079 (7)	0.0047 (6)	0.0042 (7)
N4	0.0170 (8)	0.0188 (8)	0.0148 (8)	0.0075 (7)	0.0047 (6)	0.0063 (7)
C1	0.0172 (9)	0.0187 (10)	0.0142 (9)	0.0092 (8)	0.0048 (8)	0.0044 (8)
C2	0.0159 (9)	0.0184 (9)	0.0137 (9)	0.0085 (8)	0.0063 (7)	0.0045 (8)
C3	0.0210 (10)	0.0203 (10)	0.0154 (9)	0.0075 (8)	0.0057 (8)	0.0081 (8)
C4	0.0232 (10)	0.0211 (10)	0.0157 (9)	0.0089 (9)	0.0059 (8)	0.0105 (8)
C5	0.0253 (11)	0.0285 (12)	0.0272 (12)	0.0122 (10)	0.0077 (9)	0.0132 (10)
C6	0.0341 (13)	0.0444 (15)	0.0382 (14)	0.0254 (12)	0.0152 (11)	0.0214 (12)
C7	0.0557 (17)	0.0392 (14)	0.0356 (14)	0.0350 (14)	0.0235 (13)	0.0205 (12)
C8	0.0507 (16)	0.0233 (12)	0.0286 (12)	0.0176 (11)	0.0123 (11)	0.0132 (10)
C9	0.0300 (12)	0.0226 (11)	0.0254 (11)	0.0093 (10)	0.0073 (9)	0.0132 (9)
C10	0.0178 (9)	0.0186 (9)	0.0135 (9)	0.0112 (8)	0.0050 (7)	0.0050 (8)
C11	0.0166 (9)	0.0185 (10)	0.0157 (9)	0.0096 (8)	0.0055 (8)	0.0042 (8)
C12	0.0222 (10)	0.0216 (10)	0.0167 (10)	0.0103 (9)	0.0068 (8)	0.0073 (8)
C13	0.0204 (10)	0.0189 (10)	0.0223 (11)	0.0071 (8)	0.0092 (8)	0.0061 (8)
C14	0.0147 (9)	0.0200 (10)	0.0156 (9)	0.0066 (8)	0.0041 (8)	−0.0004 (8)
C15	0.0189 (10)	0.0240 (11)	0.0137 (9)	0.0111 (9)	0.0046 (8)	0.0028 (8)
C16	0.0180 (9)	0.0184 (10)	0.0162 (9)	0.0076 (8)	0.0057 (8)	0.0048 (8)
C17	0.0126 (9)	0.0200 (10)	0.0147 (9)	0.0054 (8)	0.0041 (7)	0.0049 (8)
C18	0.0140 (9)	0.0180 (9)	0.0158 (9)	0.0055 (8)	0.0045 (7)	0.0044 (8)
C19	0.0316 (12)	0.0299 (12)	0.0154 (10)	0.0175 (10)	0.0070 (9)	0.0084 (9)
C20	0.0339 (12)	0.0318 (12)	0.0249 (11)	0.0210 (11)	0.0098 (10)	0.0151 (10)
C21	0.0196 (10)	0.0180 (10)	0.0234 (11)	0.0083 (8)	0.0076 (8)	0.0067 (8)
C22	0.0150 (9)	0.0197 (10)	0.0166 (9)	0.0057 (8)	0.0043 (7)	0.0052 (8)
C23	0.0131 (9)	0.0191 (10)	0.0153 (9)	0.0052 (8)	0.0024 (7)	0.0054 (8)
C24	0.076 (3)	0.099 (4)	0.109 (4)	0.038 (3)	0.040 (3)	0.068 (3)

### Geometric parameters (Å, °)

**Table:**

V1—O5	1.5905 (15)	C6—H6A	0.9500
V1—N4	1.8797 (17)	C7—C8	1.385 (4)
V1—O3	1.9697 (15)	C7—H7	0.9500
V1—O1	2.0036 (15)	C8—C9	1.389 (4)
V1—N1	2.0791 (17)	C8—H8	0.9500
V1—O4	2.2149 (15)	C9—H9	0.9500
Cl1—C14	1.744 (2)	C10—C11	1.474 (3)
Cl2—C21	1.736 (2)	C11—C16	1.395 (3)
O1—C1	1.310 (3)	C11—C12	1.399 (3)
O2—C1	1.217 (3)	C12—C13	1.387 (3)
O3—C10	1.299 (2)	C12—H12	0.9500
O4—C17	1.248 (2)	C13—C14	1.379 (3)
O6—C24	1.295 (6)	C13—H13	0.9500
O6—H6	0.8400	C14—C15	1.392 (3)
N1—C2	1.284 (3)	C15—C16	1.383 (3)
N1—N2	1.376 (2)	C15—H15	0.9500
N2—C10	1.318 (3)	C16—H16	0.9500
N3—C17	1.342 (3)	C17—C18	1.478 (3)
N3—N4	1.359 (2)	C18—C19	1.395 (3)
N3—H3	0.8800	C18—C23	1.395 (3)
N4—H4	0.8800	C19—C20	1.386 (3)
C1—C2	1.507 (3)	C19—H19	0.9500
C2—C3	1.493 (3)	C20—C21	1.393 (3)
C3—C4	1.525 (3)	C20—H20	0.9500
C3—H3A	0.9900	C21—C22	1.382 (3)
C3—H3B	0.9900	C22—C23	1.389 (3)
C4—C9	1.393 (3)	C22—H22	0.9500
C4—C5	1.395 (3)	C23—H23	0.9500
C5—C6	1.391 (4)	C24—H24A	0.9800
C5—H5	0.9500	C24—H24B	0.9800
C6—C7	1.385 (4)	C24—H24C	0.9800
			
O5—V1—N4	93.72 (8)	C7—C8—H8	119.9
O5—V1—O3	97.65 (7)	C9—C8—H8	119.9
N4—V1—O3	108.53 (7)	C8—C9—C4	120.5 (2)
O5—V1—O1	98.01 (7)	C8—C9—H9	119.7
N4—V1—O1	96.05 (7)	C4—C9—H9	119.7
O3—V1—O1	149.78 (6)	O3—C10—N2	123.85 (18)
O5—V1—N1	112.76 (7)	O3—C10—C11	117.81 (18)
N4—V1—N1	152.97 (7)	N2—C10—C11	118.34 (18)
O3—V1—N1	74.45 (6)	C16—C11—C12	119.72 (19)
O1—V1—N1	75.69 (6)	C16—C11—C10	120.61 (19)
O5—V1—O4	167.39 (7)	C12—C11—C10	119.66 (19)
N4—V1—O4	73.68 (7)	C13—C12—C11	120.1 (2)
O3—V1—O4	86.92 (6)	C13—C12—H12	119.9
O1—V1—O4	83.33 (6)	C11—C12—H12	119.9
N1—V1—O4	79.76 (6)	C14—C13—C12	119.0 (2)
C1—O1—V1	119.51 (13)	C14—C13—H13	120.5
C10—O3—V1	116.22 (13)	C12—C13—H13	120.5
C17—O4—V1	112.25 (13)	C13—C14—C15	121.97 (19)
C24—O6—H6	109.5	C13—C14—Cl1	119.34 (17)
C2—N1—N2	122.37 (17)	C15—C14—Cl1	118.69 (16)
C2—N1—V1	118.81 (14)	C16—C15—C14	118.7 (2)
N2—N1—V1	118.25 (13)	C16—C15—H15	120.7
C10—N2—N1	106.72 (16)	C14—C15—H15	120.7
C17—N3—N4	114.23 (16)	C15—C16—C11	120.4 (2)
C17—N3—H3	122.9	C15—C16—H16	119.8
N4—N3—H3	122.9	C11—C16—H16	119.8
N3—N4—V1	121.55 (14)	O4—C17—N3	117.01 (19)
N3—N4—H4	119.2	O4—C17—C18	123.37 (19)
V1—N4—H4	119.2	N3—C17—C18	119.62 (18)
O2—C1—O1	124.82 (19)	C19—C18—C23	119.9 (2)
O2—C1—C2	121.66 (19)	C19—C18—C17	117.78 (19)
O1—C1—C2	113.50 (18)	C23—C18—C17	122.34 (19)
N1—C2—C3	126.31 (18)	C20—C19—C18	120.3 (2)
N1—C2—C1	111.75 (18)	C20—C19—H19	119.8
C3—C2—C1	121.66 (18)	C18—C19—H19	119.8
C2—C3—C4	108.71 (16)	C19—C20—C21	118.8 (2)
C2—C3—H3A	109.9	C19—C20—H20	120.6
C4—C3—H3A	109.9	C21—C20—H20	120.6
C2—C3—H3B	109.9	C22—C21—C20	121.8 (2)
C4—C3—H3B	109.9	C22—C21—Cl2	118.73 (17)
H3A—C3—H3B	108.3	C20—C21—Cl2	119.44 (17)
C9—C4—C5	118.9 (2)	C21—C22—C23	118.96 (19)
C9—C4—C3	120.8 (2)	C21—C22—H22	120.5
C5—C4—C3	120.2 (2)	C23—C22—H22	120.5
C6—C5—C4	120.4 (2)	C22—C23—C18	120.22 (19)
C6—C5—H5	119.8	C22—C23—H23	119.9
C4—C5—H5	119.8	C18—C23—H23	119.9
C7—C6—C5	120.1 (2)	O6—C24—H24A	109.5
C7—C6—H6A	119.9	O6—C24—H24B	109.5
C5—C6—H6A	119.9	H24A—C24—H24B	109.5
C8—C7—C6	119.9 (2)	O6—C24—H24C	109.5
C8—C7—H7	120.0	H24A—C24—H24C	109.5
C6—C7—H7	120.0	H24B—C24—H24C	109.5
C7—C8—C9	120.1 (2)		
			
O5—V1—O1—C1	−118.37 (15)	C2—C3—C4—C5	83.8 (2)
N4—V1—O1—C1	147.00 (15)	C9—C4—C5—C6	−1.2 (3)
O3—V1—O1—C1	2.2 (2)	C3—C4—C5—C6	−178.3 (2)
N1—V1—O1—C1	−6.82 (14)	C4—C5—C6—C7	0.0 (4)
O4—V1—O1—C1	74.27 (14)	C5—C6—C7—C8	0.9 (4)
O5—V1—O3—C10	105.44 (15)	C6—C7—C8—C9	−0.6 (4)
N4—V1—O3—C10	−158.01 (14)	C7—C8—C9—C4	−0.5 (4)
O1—V1—O3—C10	−15.2 (2)	C5—C4—C9—C8	1.4 (3)
N1—V1—O3—C10	−6.14 (14)	C3—C4—C9—C8	178.5 (2)
O4—V1—O3—C10	−86.36 (14)	V1—O3—C10—N2	8.3 (3)
O5—V1—O4—C17	−8.3 (4)	V1—O3—C10—C11	−171.56 (13)
N4—V1—O4—C17	−9.75 (14)	N1—N2—C10—O3	−4.2 (3)
O3—V1—O4—C17	−120.08 (14)	N1—N2—C10—C11	175.61 (16)
O1—V1—O4—C17	88.58 (14)	O3—C10—C11—C16	−168.54 (18)
N1—V1—O4—C17	165.18 (15)	N2—C10—C11—C16	11.6 (3)
O5—V1—N1—C2	100.74 (16)	O3—C10—C11—C12	12.4 (3)
N4—V1—N1—C2	−67.0 (2)	N2—C10—C11—C12	−167.50 (19)
O3—V1—N1—C2	−167.36 (16)	C16—C11—C12—C13	−0.3 (3)
O1—V1—N1—C2	7.94 (15)	C10—C11—C12—C13	178.82 (18)
O4—V1—N1—C2	−77.72 (15)	C11—C12—C13—C14	0.9 (3)
O5—V1—N1—N2	−87.67 (15)	C12—C13—C14—C15	−0.5 (3)
N4—V1—N1—N2	104.63 (19)	C12—C13—C14—Cl1	179.08 (16)
O3—V1—N1—N2	4.23 (13)	C13—C14—C15—C16	−0.6 (3)
O1—V1—N1—N2	179.52 (14)	Cl1—C14—C15—C16	179.84 (16)
O4—V1—N1—N2	93.86 (14)	C14—C15—C16—C11	1.2 (3)
C2—N1—N2—C10	169.75 (18)	C12—C11—C16—C15	−0.8 (3)
V1—N1—N2—C10	−1.5 (2)	C10—C11—C16—C15	−179.91 (18)
C17—N3—N4—V1	−8.5 (2)	V1—O4—C17—N3	8.5 (2)
O5—V1—N4—N3	−170.15 (15)	V1—O4—C17—C18	−170.65 (15)
O3—V1—N4—N3	90.49 (15)	N4—N3—C17—O4	−1.4 (3)
O1—V1—N4—N3	−71.68 (15)	N4—N3—C17—C18	177.84 (17)
N1—V1—N4—N3	−1.5 (3)	O4—C17—C18—C19	−4.5 (3)
O4—V1—N4—N3	9.53 (14)	N3—C17—C18—C19	176.35 (19)
V1—O1—C1—O2	−173.47 (16)	O4—C17—C18—C23	174.85 (19)
V1—O1—C1—C2	5.1 (2)	N3—C17—C18—C23	−4.3 (3)
N2—N1—C2—C3	−4.7 (3)	C23—C18—C19—C20	−1.3 (3)
V1—N1—C2—C3	166.52 (16)	C17—C18—C19—C20	178.0 (2)
N2—N1—C2—C1	−178.68 (16)	C18—C19—C20—C21	−0.3 (4)
V1—N1—C2—C1	−7.5 (2)	C19—C20—C21—C22	1.6 (4)
O2—C1—C2—N1	−179.70 (19)	C19—C20—C21—Cl2	−177.59 (19)
O1—C1—C2—N1	1.7 (2)	C20—C21—C22—C23	−1.2 (3)
O2—C1—C2—C3	6.0 (3)	Cl2—C21—C22—C23	178.00 (15)
O1—C1—C2—C3	−172.65 (18)	C21—C22—C23—C18	−0.5 (3)
N1—C2—C3—C4	−93.3 (2)	C19—C18—C23—C22	1.8 (3)
C1—C2—C3—C4	80.1 (2)	C17—C18—C23—C22	−177.57 (18)
C2—C3—C4—C9	−93.2 (2)		

### Hydrogen-bond geometry (Å, °)

**Table:**

*D*—H···*A*	*D*—H	H···*A*	*D*···*A*	*D*—H···*A*
N3—H3···O1^i^	0.88	1.98	2.741 (2)	143
N4—H4···O6^i^	0.88	1.94	2.792 (3)	162
O6—H6···O2	0.84	2.27	2.908 (3)	133

Symmetry codes: (i) −*x*+1, −*y*+1, −*z*+1.
